# Wall characteristics of atherosclerotic middle cerebral arteries in patients with single or multiple infarcts: A high-resolution MRI Study

**DOI:** 10.3389/fneur.2022.934926

**Published:** 2022-11-03

**Authors:** Zelan Ma, Mengjuan Huo, Jiajun Xie, Guoqing Liu, Guoming Li, Qiang Liu, Liting Mao, Weikang Huang, Bo Liu, Xian Liu

**Affiliations:** ^1^Department of Radiology, The Second Affiliated Hospital of Guangzhou University of Chinese Medicine, Guangzhou, China; ^2^Department of Radiology, Guangzhou First People's Hospital, School of Medicine, South China University of Technology, Guangzhou, China; ^3^Department of Neurosurgery, The Second Affiliated Hospital of Guangzhou University of Chinese Medicine, Guangzhou, China

**Keywords:** high-resolution magnetic resonance imaging (HRMRI), diffusion-weighted imaging (DWI), atherosclerosis, stroke, histogram

## Abstract

**Background and purpose:**

Understanding the stroke mechanism of middle cerebral artery (MCA) atherosclerosis may inform secondary prevention. The aim of this study was to explore the relationship between vascular wall characteristics and infarction patterns using high-resolution magnetic resonance imaging (HRMRI) and diffusion-weighted imaging (DWI).

**Methods:**

From November 2018 to March 2021, patients with acute ischemic stroke due to MCA atherosclerotic disease were retrospectively analyzed. The wall characteristics of atherosclerotic MCA, including conventional characteristics and histogram-defined characteristics, were evaluated using HRMRI. Patients were divided into single-infarction and multiple-infarction groups based on DWI, and wall characteristics were compared between the two groups.

**Results:**

Of 92 patients with MCA plaques, 59 patients (64.1%) had multiple infarcts, and 33 (35.9%) had single infarcts. The histogram-defined characteristics showed no differences between the single-infarction and multiple-infarction groups (*P*>0.05). Plaque burden, degree of stenosis, and prevalence of intraplaque hemorrhage (IPH) were significantly greater in the multiple-infarction group than in the single-infarction group (plaque burden: *P* = 0.001; degree of stenosis: *P* = 0.010; IPH: *P* = 0.019). Multivariate analysis showed that plaque burden (odds ratio: 1.136; 95% confidence interval: 1.054–1.224, *P* = 0.001) and IPH (odds ratio: 5.248; 95% confidence interval: 1.573–17.512, *P* = 0.007) were independent predictors for multiple infarction.

**Conclusion:**

IPH and plaque burden are independently associated with multiple infarcts. HRMRI may provide new insight into the mechanisms underlying the different MCA infarction patterns.

## Introduction

Intracranial large artery atherosclerosis (LAA) accounts for almost 33–50% of ischemic strokes in the Chinese population and is associated with a high risk of ischemic event recurrence (1, 2). Previous studies have shown that patients with multiple infarcts due to LAA have a higher rate of recurrent stroke than those with single infarcts, even though the former received more clinical benefits from dual antiplatelet therapy (3–5). Further understanding the difference in the underlying vascular pathophysiology between different infarction patterns could be helpful for stroke risk stratification and treatment strategy modification.

Recently, high-resolution magnetic resonance imaging (HRMRI) has shown the ability to disclose the underlying pathomechanism of LAA with different infarction patterns by detailed plaque characteristics such as plaque location, morphology, and components (6–8). For example, HRMRI-defined intraplaque hemorrhage (IPH) and plaque surface irregularity were more frequently observed in artery-to-artery embolic infarction than in perforating artery infarction (9).

Moreover, novel plaque characterization techniques with HRMRI, such as quantitative histogram analysis, have attracted increased attention in recent years. Histogram analysis is an objective method that could reflect the distribution of signal intensity in a region of interest. Pilot studies have reported that this approach could provide more abundant information for the identification of plaque types (10, 11). In the study conducted by Shi et al. (10), the authors found that compared with conventional plaque characteristics such as IPH and plaque burden, parameters derived from histograms can improve the differentiation between culprit and non-culprit plaques in the intracranial artery. However, this approach has not yet been applied to explore the characteristics of intracranial culprit plaques ultimately leading to the different number of infarcts.

Therefore, in this study, HRMRI was used to determine the difference in the conventional and histogram-defined characteristics of the culprit plaques between the patients with multiple infarcts and those with a single infarct.

## Methods

### Subjects

Our hospital's Ethics Committees approved this study, and written informed consent was waived because this was a retrospective study. Patients were consecutively collected in this study from November 2018 to March 2021 if they fulfilled the following criteria: (1) an acute ischemic stroke in the middle cerebral artery (MCA) territory identified by diffusion-weighted imaging (DWI) within 7 days of symptom onset; (2) underwent intracranial HRMRI within 14 days after admission; (3) evidence of atherosclerotic plaque detected by HRMRI in the M1 segment of the MCA; (4) absence of coexistent carotid artery or anterior cerebral artery stenosis (≥50%) on MRA; (5) no evidence of stroke caused by non-atherosclerotic vascular diseases such as dissection, vasculitis, and cardiogenic embolism; and (6) no evidence of cardiogenic embolism (12), such as recent myocardial infarction <4 weeks, mitral stenosis or prosthetic valve, patent foramen ovale, atrial fibrillation, acute bacterial endocarditis, and so on. Total occlusion of MCA was excluded from this study. Clinical information was recorded for each patient, including age, sex, National Institute of Health Stroke Scale (NIHSS) score at admission, and vascular risk factors, including hypertension, diabetes mellitus, hyperlipidemia, current smoking, previous coronary artery disease, and previous cerebrovascular disease.

### Infarction pattern

According to the number of hyperintense lesions in the MCA territory detected on the DWI images, patients were divided into single-infarction and multiple-infarction groups. Multiple infarcts refer to more than one non-contiguous lesion occurring in atherosclerotic MCA territory.

### MRI protocols

Conventional brain MRI was initially performed for clinical evaluation of stroke patients; the imaging sequences included T1-weighted, T2-weighted, fluid-attenuated inversion recovery, and DWI. Imaging parameters for DWI were TR/TE = 4,040/55 ms, field of view = 220 × 220 mm^2^, matrix = 160 × 160, slice thickness = 5 mm, 2 b values of 0 and 1,000 s/mm^2^. All patients underwent the HRMRI using a 3T MR scanner (MAGNETOM Prisma, Siemens Healthineers) with a 64-channel head coil. The HRMRI protocol consisted of three-dimensional time-of-flight MR angiography (3D TOF-MRA), 2D T2-weighted vessel wall sequence, precontract and postcontrast 3D T1-weighted sampling perfection with application-optimized contrasts using different flip angle evolutions (3D T1W-SPACE) sequence. 3D TOF-MRA is mainly used to assess arterial lumen condition. 2D T2-weighted vessel wall sequence was performed perpendicular to the MCA M1 segment after 3D TOF-MRA. The HRMRI scanning parameters for MCA were follows: (1) 3D TOF MRA: TR/TE = 21/3.42 ms, field of view = 200 × 175 mm^2^, slice thickness = 0.55 mm, NEX = 1, matrix = 319 × 384, sequence duration = 4 min 49 s; (2) 2D T2-weighted image: TR/TE = 3,770/62 ms, field of view = 150 × 150 mm^2^, 12 slices, matrix = 384 × 384, echo train length = 18, slice thickness = 2 mm, and scan time = 3 min 40 s; (3) 3D T1W-SPACE: sagittal orientation, TR/TE = 800/19 ms, field of view = 207 × 178 mm^2^, 256 slices, matrix = 384 × 330, voxel size = 0.54 × 0.54 × 0.54 mm^3^, echo train length = 52, and scan time = 9 min, 30 s. Contrast-enhanced 3D SPACE was repeated 5 min after administering 0.1 mmol/kg contrast agent (Magnevist; Schering, Berlin, Germany).

### Conventional HRMRI characterization analysis

The HRMRI images were analyzed using the IntelliSpace Portal workstation (Philips Medical Systems, the Netherlands). Image quality was evaluated using a 3-point scale (1 = poor, 2 = adequate, 3 = good) and cases with an image quality score of 1 were excluded. A culprit plaque was defined as a lesion within the vascular territory of the stroke on DWI with accompanying clinical symptoms (13). The maximal-lumen-narrowing (MLN) site and reference site of MCA (proximal to the lesion site) were evaluated, respectively, and cross-sectional views perpendicular to the MCA long axis were reconstructed on postcontrast 3D T1W-SPACE images for quantitative morphological measurements, including maximal wall thickness (WT), lumen area (LA), and vessel area (VA). Plaque area and plaque burden were measured on the MLN site, plaque area was calculated as (VA_MLN_–LA_MLN_), and plaque burden was defined as (Plaque area / VA_MLN_) × 100%. The stenosis value was calculated as (1—LA_MLN_ / LA_reference_) × 100%. Remodeling index (RI) was calculated as VA_MLN_ / VA_reference_. RI ≥ 1.05 was defined as positive remodeling (PR), 0.95 < RI < 1.05 as intermediate remodeling (IR), and RI ≤ 0.95 as negative remodeling (NR). Patterns of the remodeling were divided into two groups, namely, PR and non-PR (IR and NR) (14).

Plaque geometry features include eccentricity and concentricity. Eccentricity was defined as a localized plaque surrounding < 75% of the vessel wall, or the maximal WT being two times larger than the minimal WT (15). The plaque location was described using a “4-arc” standard according to previous studies. It was classified into perforator arc (plaque involved superior and/or dorsal wall where perforator originated from) and non-perforator arc (16). IPH was characterized by an area of high signal (>150% of the signals of adjacent gray matter) within the plaque on the T1WI sequence (17). Plaque surface irregularity was defined as a discontinuity of the plaque surface on contrast-enhanced T1-weighted images. Plaque enhancement was defined as an enhancement degree of the culprit plaque higher than that of the normal adjacent vessel wall (18).

### Histogram-defined characteristic analysis

T2-weighted images in DICOM format were extracted from the institutional PACS and imported into the open-source software Image J (National Institutes of Health, Bethesda, MD) for histogram analysis. Regions of interest were manually drawn as precisely as possible along the edge of the plaque in the MLN site of MCA. Then, Image J calculated the following voxel-based histogram parameters automatically for the ROIs: mean, standard deviation (SD), minimum, maximum, median, skewness, kurtosis, and coefficient of variation (CV). The CV was calculated as SD/mean. The skewness describes the degree of asymmetry based on the average. The kurtosis describes the flatness or peak of the curve. The CV describes the dispersion of signal intensity (10).

### Interobserver reproducibility analysis

To assess interobserver reproducibility, two radiologists (ZLM and MJH, with 5 and 7 years of experience in the diagnostics of neuroradiology, respectively) blinded to the patient's clinical data analyzed the qualitative HRMRI features independently, including plaque eccentricity, vessel remodeling pattern, IPH, plaque enhancement, plaque location, and plaque surface irregularity. In situations of disagreement, a third assessor (JJX) adjudicated.

For quantitative HRMRI features including plaque area, plaque burden, plaque length, maximal WT and degree of stenosis, mean, SD, minimum, maximum, median, skewness, kurtosis, and CV, a subgroup of 30 cases of each group was randomly selected and reassessed by two radiologists (ZLM and JJX) independently.

### Statistical analysis

All statistical analyses were performed using the SPSS 22.0 statistical software. The interclass correlation coefficient (ICC) (19) was used to evaluate the interobserver reproducibility of the quantitative HRMRI features. ICC values that were ≥0.75 were considered good agreement. The kappa value was used to evaluate the interobserver agreement for the qualitative HRMRI features. A kappa value of 0.00–0.20 indicates poor agreement, 0.21–0.40 is fair, 0.41–0.60 is moderate, 0.61–0.80 is good, and 0.81–1.00 is excellent. The normality of the data was evaluated using the Shapiro-Wilk test. Continuous variables were described as the mean ± standard deviation or median and interquartile range (if not normally distributed). Continuous data were compared using either a Student's *t*-test or a Mann–Whitney U test (if not normally distributed). Categorical variables were listed as numbers and percentages (%) and were compared using the chi-square test or Fisher's exact test. For predicting multiple infarcts on DWI, parameters with *P*-values < 0.1 from univariate analysis were included in the following multivariate logistic regression analysis. A value of two-tailed *P* < 0.05 was defined as statistical significance.

## Results

### Patient characteristics

A total of 92 consecutive patients (67 males and 25 females) who met the inclusion criteria were finally enrolled. Of the 92 patients, 33 (35.9%) were identified in the single-infarction group, and 59 (64.1%) were identified in the multiple-infarction group. There were no significant differences in the clinical characteristics between the two groups (Table 1).

**Table 1:** Clinical characteristics of patients with single infarction and multiple infarction.

	**Single-infarction**	**Multiple-infarction**	***P*-value**
	**group (*n* = 33)**	**group (*n* = 59)**	
Age (mean ± SD, y)	57.94 ± 14.16	59.02 ± 13.92	0.724
Male sex, *n (%)*	24 (72.7%)	43 (72.9%)	0.586
Hypertension, *n (%)*	19 (57.6%)	31 (52.5%)	0.403
Diabetes mellitus, *n (%)*	6 (18.2%)	17 (28.8%)	0.191
Hyperlipidemia, *n (%)*	4 (12.1%)	13 (22.0%)	0.240
Current smoking, *n (%)*	11 (33.3%)	27 (45.8%)	0.174
NIHSS score, median (P25, P75)	2 (1, 6)	4 (2, 6)	0.061
History of CAD	3 (9.1%)	5 (8.5%)	0.600
History of CVD	2 (6.1%)	4 (6.8%)	0.632

NIHSS, National Institutes of Health Stroke Scale score; P25, 1st quartile; P75, 3rd quartile; CAD, coronary artery disease; CVD, cerebrovascular disease.

### MR imaging measurements reproducibility

Interobserver reproducibility was good for measuring conventional quantitative HRMRI characteristics and histogram parameters. The ICCs of the conventional features for the two reviewers in measuring the plaque area, plaque burden, plaque length, maximal WT, and degree of stenosis were 0.865 (95% CI: 0.733–0.934) and 0.853 (95% CI: 0.704–0.928), 0.970 (95% CI: 0.922–0.987), 0.942 (95% CI: 0.882–0.972), and 0.912 (95% CI: 0.823–0.957), respectively. The ICCs of the histogram parameters for the two reviewers in measuring the mean, SD, minimum, maximum, median, skewness, kurtosis, and CV were 0.984 (95% CI: 0.967–0.993), 0.895 (95% CI: 0.780–0.950), 0.948 (95% CI: 0.890–0.975), 0.934 (95% CI: 0.860–0.968), 0.981 (95% CI: 0.960–0.991), 0.822 (95% CI: 0.626–0.915), 0.825 (95% CI: 0.630–0.916), and 0.918 (95% CI: 0.828–0.961), respectively.

Interobserver agreement was excellent for the identification of plaque eccentricity (kappa value, 0.870), remodeling pattern (kappa value, 0.847), IPH (kappa value, 0.881), and plaque enhancement (kappa value, 0.903). Interobserver agreement was good for the identification of plaque location (kappa value, 0.799) and plaque surface irregularity (kappa value, 0.739).

### Comparison of HRMRI-derived characteristics between the two groups

**Figure 1:**
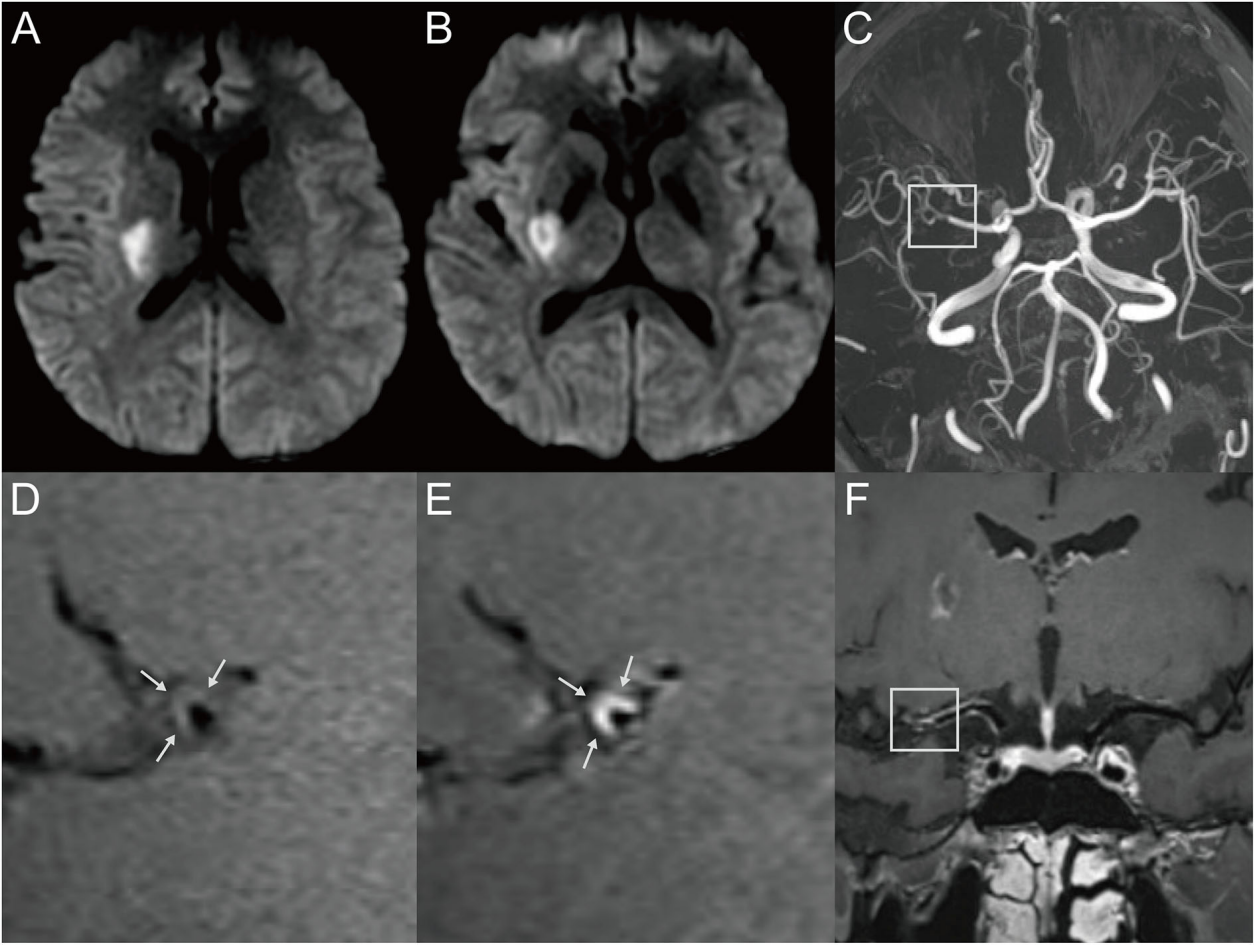
Representative MR images of a 34-year-old male patient with a single acute infarct lesion. The patient presented with left-side weakness for 2 days. The patient's risk factors included hypertension and hyperlipidemia. **(A,B)** The DWI images showed an infarct in the right basal ganglion region. **(C)** The 3D-TOF-MRA showed moderate stenosis of the right middle cerebral artery (MCA) (box). **(D)** The T1-weighted HRMRI demonstrated an eccentric atherosclerotic plaque with iso-signal intensity at the corresponding location (arrow), indicating probable parent artery atherosclerosis occluding a penetrating artery. **(E)** Postcontrast T1-weighted HRMRI showed strong enhancement of the plaque (arrow). **(F)** The postcontrast T1-weighted image by curved planar reformation showed the location of the plaque, which involved the superior wall where the perforator originated (box).

**Figure 2:**
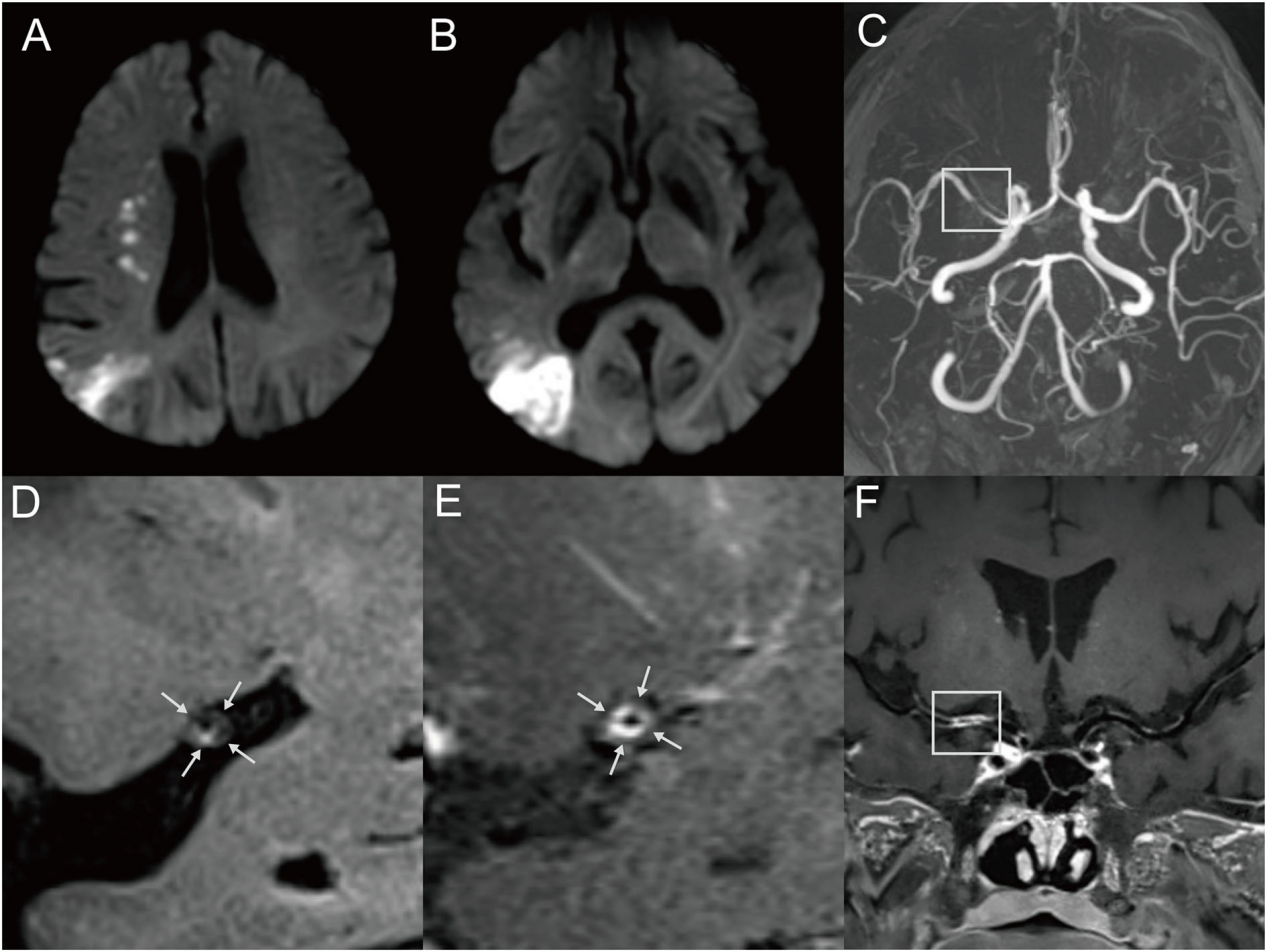
Representative MR images of a 60-year-old male patient with multiple MCA infarctions. **(A,B)** DWI showed chain-like internal border-zone infarctions and wedge-shaped or spotty cortical infarctions in the right MCA territory. The patient with a cigarette smoking history presented with left-side weakness and mouth askew for 4 days. **(C)** 3D-TOF-MRA demonstrated severe stenosis (box) of the right MCA. **(D)** Pre-contrast HRMRI showed a diffuse distributive plaque with a spotted high signal (intraplaque hemorrhage) at the inferior wall of MCA (arrow). **(E)** Postcontrast HRMRI demonstrated obvious plaque enhancement (arrow). **(F)** The postcontrast T1-weighted image by curved planar reformation showed a diffuse plaque located at the proximal M1 segment of the right MCA (box).

The conventional HRMRI characteristics of the culprit plaques on MCA are summarized in Table 2. Plaque burden and degree of stenosis were significantly greater in the multiple-infarction group than in the single-infarction group (plaque burden: *P* = 0.001; degree of stenosis: *P* = 0.010). Furthermore, intraplaque hemorrhage was more frequently observed in the multiple-infarction group (*P* = 0.019). Representative cases are illustrated in Figures 1, 2. No significant difference was found between the two groups in plaque area, plaque length, maximum WT, eccentricity, plaque location, remodeling pattern, plaque surface irregularity, or enhancement degree.

**Table 2:** Conventional HRMRI characteristics between single-infarction and multiple-infarction groups.

	**Single-infarction**	**Multiple-infarction**	***P*-value**
	**group (*n* = 33)**	**group (*n* = 59)**	
Plaque area, mm^2^	10.04 (7.91, 11.65)	10.40 (8.35, 12.87)	0.183
Plaque burden, %	87.44 (77.34, 92.61)	91.29 (89.35, 94.26)	0.001
Plaque length, mm	6.80 (4.35, 10.85)	8.30 (5.30, 10.20)	0.159
Maximum WT, mm	1.70 (1.50, 1.85)	1.60 (1.30, 1.90)	0.169
Eccentricity	20 (60.6%)	27 (45.8%)	0.172
Plaque location			0.162
Perforator arc	20 (60.6%)	44 (74.6%)	
Non-perforator arc	13 (39.4%)	15 (25.4%)	
Remodeling Pattern			0.398
PR	16 (48.5%)	34 (57.6%)	
Non-PR	17 (51.5%)	25 (42.4%)	
Intraplaque hemorrhage	6 (18.2%)	25 (42.4%)	0.019
Plaque surface irregularity	14 (42.4%)	29 (49.2%)	0.535
Enhancement pattern			0.346
Non-enhancement	3 (9.1%)	2 (3.4%)	
Enhancement	30 (90.9%)	57 (96.6%)	
Degree of stenosis, %	66.19 (52.78, 78.88)	76.33 (65.33, 83.28)	0.010

WT, wall thickness; PR, positive remodeling.

**Figure 3:**
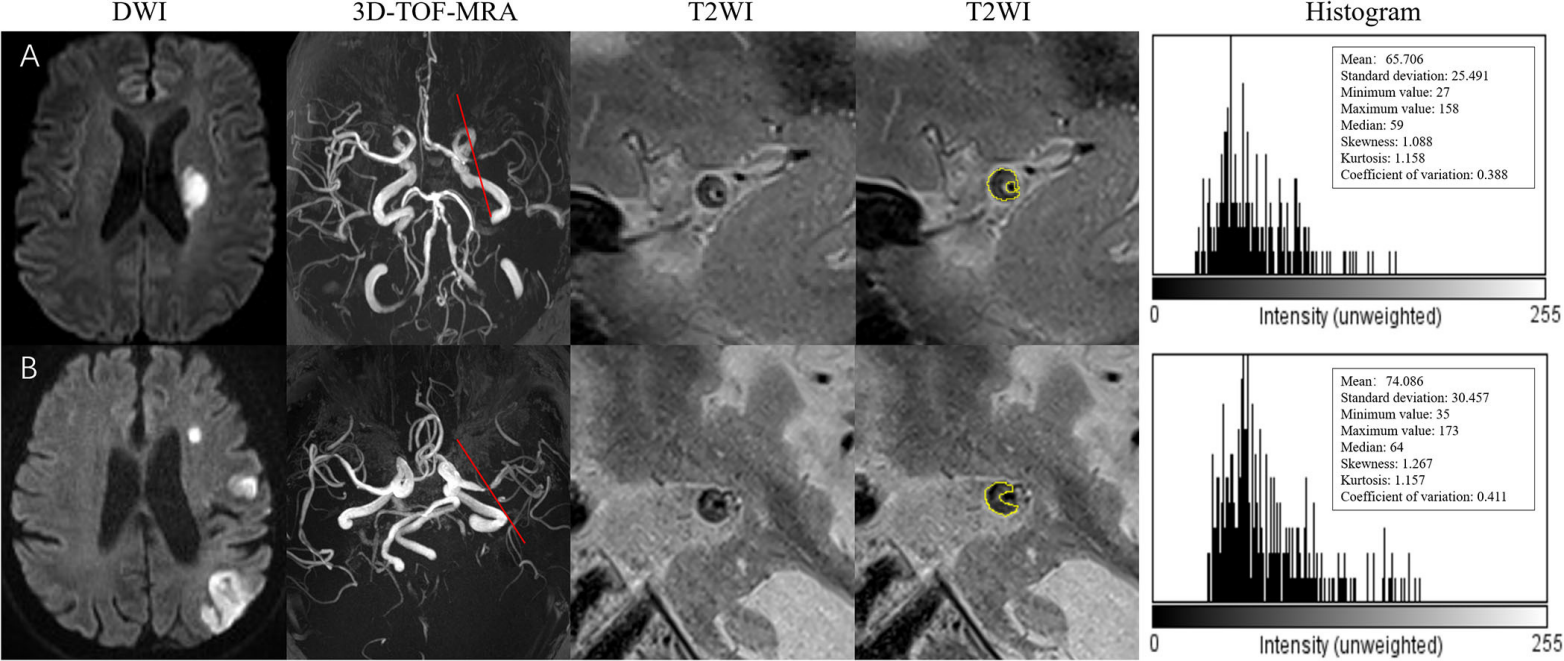
**(A)** A 73-year-old man presented with right-side weakness for 3 days. DWI showed an oval acute infarct in the left corona radiate. 3D-TOF-MRA demonstrated the stenosis located on the left MCA. The T2-weighted HRMRI demonstrated an eccentric atherosclerotic plaque with heterogeneity intensity at the most stenotic site of the right MCA. The outer wall boundary of the plaque can be outlined. The quantitative histogram parameters based on T2WI are shown on the right. **(B)** A 59-year-old man presented with right-side weakness and speech disorder for 5 days. DWI showed multiple infarctions in the left MCA territory. 3D-TOF-MRA demonstrated the stenosis located on the left MCA. The T2-weighted HRMRI demonstrated an eccentric atherosclerotic plaque with heterogeneity intensity at the most stenotic site of the right MCA. The outer wall boundary of the plaque can be outlined. The quantitative histogram parameters based on T2WI are shown on the right.

Histogram-defined characteristics are presented in Table 3 with representative cases shown in Figure 3. Univariate analysis showed no significant differences between the two groups in histogram-defined characteristics, including mean, SD, minimum, maximum, median, skewness, kurtosis, or CV.

**Table 3:** Histogram features between single-infarction and multiple-infarction groups.

	**Single-infarction**	**Multiple-infarction**	***P*-value**
	**group (*n* = 33)**	**group (*n* = 59)**	
Mean	93.97 ± 26.83	102.87 ± 20.17	0.065
SD	24.02 ± 8.74	25.58 ± 9.93	0.209
Minimum	44.39 ± 27.14	51.86 ± 22.31	0.138
Maximum	153.82 ± 38.41	165.71 ± 36.50	0.125
Median	93.33 ± 28.14	103.12 ± 23.38	0.063
Skewness	0.26 ± 0.51	0.18 ± 0.47	0.470
Kurtosis	0.01 ± 0.84	−0.16 ± 0.67	0.379
CV	0.27 ± 0.12	0.26 ± 0.11	0.804

SD, standard deviation; CV, coefficient of variation.

### Multivariate logistic regression analysis

Based on the results of the univariate analysis (Tables 1–3), the variables of NIHSS score, plaque burden, IPH, degree of stenosis, mean, and median (*P* < 0.1) were used as input variables for the multivariate logistic regression analysis.

Multivariate analysis identified plaque burden as an independent predictor of multiple-infarction group (odds ratio: 1.136; 95% confidence interval: 1.054–1.224, *P*=0.001). Intraplaque hemorrhage was also independently associated with multiple-infarction group (odds ratio: 5.248; 95% confidence interval: 1.573–17.512, *P* = 0.007) (Table 4).

**Table 4:** Predictors for multiple infarction in patients with culprit plaque in the middle cerebral artery.

**Parameter**	***P*-value**	**β**	**Odds ratio**	**95% confidences**	
				**interval**	
				**Lower**	**Upper**
Plaque burden	0.001	0.127	1.136	1.054	1.224
Intraplaque hemorrhage	0.007	1.658	5.248	1.573	17.512

## Discussion

This study used a combination of HRMRI and DWI to explore the possible pathomechanism of different infarction patterns in patients with acute stroke due to MCA atherosclerosis. We found that the patients with multiple infarction were more likely to have a higher occurrence of IPH and a greater plaque burden than those with single infarction. However, there were no significant differences between the single-infarction and multiple-infarction groups for the histogram-defined plaque characteristics.

Histogram analysis is a computer-assisted methodology for extracting quantitative features that cannot be visualized from images. Histogram analysis shows promise in multiple pathologies, including glioma, thyroid nodule, and rectal cancer (20–22). However, its application has not been widely investigated in intracranial plaques with HRMRI, and all of the previous studies (10, 11, 23, 24) focus on differentiating symptomatic from asymptomatic plaques. To our knowledge, this is the first study using an HRMRI-based histogram approach to explore the features of culprit plaques that lead to different infarction patterns. In this study, there was no association between the histogram parameters of MCA plaques and infarction patterns. The following two factors may explain the phenomenon. First, the volumes of intracranial plaques were relatively small and the components of the plaques were heterogeneous. Thus, the T2-weighted intensity may be averaged or washed out if various plaque components exist in a micro distribution. Second, IPH and lipid core are important elements of vulnerable plaque, but both of them could present as hyperintensity or isointense on T2-weighted imaging (25, 26). As it lacks histological validation, it is hard to determine what proportion of T2-weighted intensity is due to specific plaque components. Therefore, multicontrast MRIs such as T1-weighted imaging need to be further studied to investigate whether they can help differentiate the plaque components.

IPH is thought to be caused by the disruption of microvessels and intraplaque vasa vasorum. IPH detected on T1-weighted HRMRI has been studied as a potential marker of stroke risk (14, 27). However, a few studies of intracranial HRMRI have concentrated on the relationship between infarct patterns and IPH (9, 28). Our study showed that IPH carried a 5.248-fold risk of multiple infarction in acute stroke patients with MCA atherosclerotic disease, indicating that the plaques causing multiple infarcts may be more vulnerable than the plaques causing a single infarct. A similar finding was reported in a previous study (9). The authors found that patients with artery-to-artery embolism had a higher frequency of IPH than perforating artery infarction, indicating the instability of plaques can present with thromboembolism events, leading to multiple cerebral infarcts. However, another study reported no association between IPH and infarct patterns. This difference may be attributable to the small sample in their study, which just includes 12 and 33 patients with single infarction and multiple infarction due to atherosclerotic MCA, respectively (28).

In this study, we found that every 10% increase in plaque burden would lead to a 2.26-fold higher risk of multiple infarction. Previous studies reported that increased plaque burden might strongly affect the patterns of cerebral hemodynamics and promote the formation of thrombi engrafted upon atherosclerotic plaques, causing hypoperfusion and artery-to-artery embolism (29, 30), which might account for our result that patients with greater plaque burden more frequently demonstrated multiple infarcts.

A smaller study investigating the characteristics of intracranial and extracranial arterial culprit plaques between patients with recent ischemic stroke showed that higher stenosis was associated with multiple infarcts (28). Their findings are in agreement with our study. However, in the multivariate analysis, the degree of stenosis was not a significant predictor of multi-infarct patterns, possibly related to vessel wall remodeling. There is increasing evidence that vessel wall characteristics provide additional value over luminal stenosis degree alone (31, 32).

A previous study suggested that plaque surface irregularity was a significant predictor of multiple acute infarcts (29). However, we draw different conclusions from this study. This difference may be attributed to different MR sequences used to evaluate the status of the plaque surface. Our study used the postcontrast instead of the pre-contrast T1WI sequence. Since HRMRI-defined plaque surface irregularity lacks histological validation, which MR sequence would be the best choice to reflect plaque surface status remains unclear, further work is needed to provide more evidence.

Our study has several limitations. First, the sample size was relatively small, yet to our knowledge, this is the largest reported series specifically focusing on the relationship between MCA plaque features and infarct patterns. Further large sample size studies are needed to verify our results. Second, the study recruited patients with strokes in the MCA territory only. Therefore, our conclusion has to be extrapolated to other large arteries, such as the basilar artery with caution. Third, because the scan time of the 3D vessel wall imaging sequence was relatively long, it is possible that clinically stable patients were included, leading to possible selection bias. Novel and efficient MR-based imaging techniques are needed to reduce scan time. Finally, our study was limited in its inability to pathological verification because of the difficulty of obtaining specimens of intracranial vessels.

## Conclusions

Multiple infarction has distinct plaque characteristics, including IPH and greater plaque burden compared with single infarction, possibly because of vulnerable plaques and hemodynamic compromise in ischemic regions. Future studies are needed to explore the mechanisms underlying the different MCA infarction patterns and guide clinical treatments.

## Data availability statement

The original contributions presented in the study are included in the article/supplementary material, further inquiries can be directed to the corresponding authors.

## Ethics statement

The studies involving human participants were reviewed and approved by the Institutional Review Board of the Second Affiliated Hospital of Guangzhou University of Chinese Medicine. The Ethics Committee waived the requirement of written informed consent for participation.

## Author contributions

ZM and MH contributed to study conception and design and drafted the manuscript. JX, GLiu, GLi, QL, LM, and WH contributed to data acquisition, data interpretation, and analysis. BL and XL critically revised the paper and approved the final version of the manuscript. All authors contributed to the article and approved the submitted version.

## Funding

This study was supported by the National Natural Science Foundation of China (No. 81801688) and Administration of Traditional Chinese Medicine of Guangdong Province of China (Nos. 20231141 and 20211173).

## Conflict of interest

The authors declare that the research was conducted in the absence of any commercial or financial relationships that could be construed as a potential conflict of interest.

## Publisher's note

All claims expressed in this article are solely those of the authors and do not necessarily represent those of their affiliated organizations, or those of the publisher, the editors and the reviewers. Any product that may be evaluated in this article, or claim that may be made by its manufacturer, is not guaranteed or endorsed by the publisher.
